# Biological and technical complications in root cap–retained overdentures after 3–15 years in situ: a retrospective clinical study

**DOI:** 10.1007/s00784-020-03555-3

**Published:** 2020-09-07

**Authors:** Anja Stalder, Camille Henriette Berger, Ramona Buser, Julia Wittneben, Martin Schimmel, Samir Abou-Ayash

**Affiliations:** 1 Department of Reconstructive Dentistry and Gerodontology, School of Dental Medicine, University of Bern, Bern, Switzerland; 2 Division of Gerodontology and Removable Prosthodontics, University of Geneva, Geneva, Switzerland

**Keywords:** Overdentures, Intraoral microbiome, Root-caps, Post-and-cores

## Abstract

**Objectives:**

This retrospective clinical study investigates the frequency of biological and technical complications in patients rehabilitated by natural root-retained overdentures (RODs) with cast post-and-cores (root caps) wearing precision attachments and analyses factors influencing complication rates (e.g. oral hygiene routines).

**Materials and methods:**

Patients formerly treated with RODs were invited for a cost-free clinical visit to evaluate their intraoral status. Furthermore, they were interviewed and patient records were screened for complications occurring since denture delivery. Statistical models include descriptive analyses, Fisher’s exact test, odds ratios, and a multivariate regression model.

**Results:**

A total of 114 patients wearing 128 RODs with a total of 280 abutment teeth were evaluated (mean service time: 7.9 years). Technical complications occurred in 68.8% of the RODs, with matrix loosening being the most frequent complication (50.1%). Biological complications occurred in 53.9% of all RODs, with the presence of denture stomatitis being the most common biological complication (38.3%). The presence of denture stomatitis was significantly higher in the maxilla relative to the mandible (*p* = 0.0029), in subjects cleaning their dentures less than twice a day (*p* < 0.001), in subjects regularly using CHX-containing products (*p* = 0.036) and in subjects with a plaque index > 40% (*p* < 0.001).

**Conclusions:**

Root cap-retained overdentures with precision attachments are a viable treatment option in partially dentate subjects, even over long-term periods. However, high complication rates should be expected.

**Clinical relevance:**

Establishing good oral hygiene is a decisive factor in preventing complications in RODs. Furthermore, CHX-containing products may not be recommended for routine domestic use.

## Introduction

Due to the continuous development of oral care and strategies and preventative measures, the frequency of caries and periodontitis has decreased over the past several decades. Consequently, the number of people suffering from total edentulism is also decreasing, whereas the number of people with residual teeth is rising [1]. However, remaining teeth usually have undergone endodontic treatment and/or exhibit reduced periodontal attachment due to continuous age-dependent bone loss, resulting in an unfavorable crown-root ratio [2, 3]. If tooth replacement with removable partial dentures (RPDs) becomes necessary, such teeth are known to make poor abutments for conventional clasp-retained RPDs, because of their increased probability of tooth loss [4]. Consequently, teeth with a reduced periodontal ligament and/or endodontic treatment are often extracted in these situations, and removable complete dentures (RCDs) are fabricated, especially when economic considerations play a significant role in the decision-making process.

One strategy to reestablish a more favorable crown-root ratio is to shorten the clinical crown. Subsequently, teeth with shortened crowns can serve as abutments in a root-supported overdenture (ROD) [5]. Overdentures are defined as removable dental prostheses that cover and are partially supported by natural teeth, natural tooth roots and/or dental implants [6]. Usually, ROD abutment teeth undergo elective endodontic treatment because of the extended shortening required to provide sufficient vertical space for prosthetic rehabilitation [7]. Nevertheless, even endodontically treated abutment teeth provide a certain degree of proprioception and sensibility, as the periodontal ligament and the according receptors are preserved [8]; this results in a higher chewing efficiency relative to patients rehabilitated by complete dentures or implant-supported overdentures [9]. Furthermore, bone resorption in patients wearing mandibular RODs is almost an order of magnitude lower than among patients wearing mandibular complete dentures [10].

**Fig. 1:**
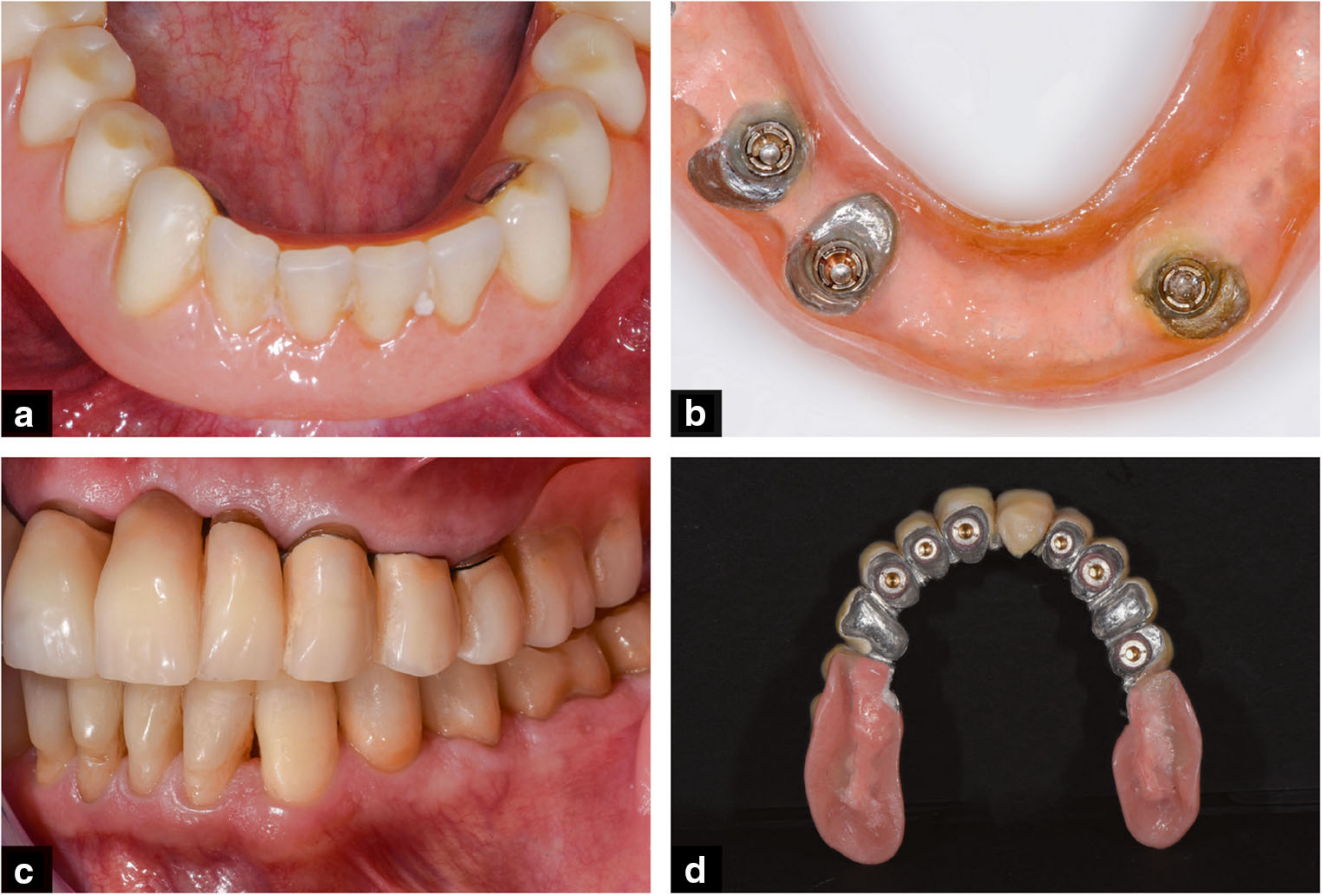
**a**, **b** Intra- and extraoral view of an ROD with a closed denture design (complete coverage of the abutment teeth). **c**, **d** Intra- and extraoral view of an ROD with an open denture design (finishing line on the abutment teeth)

To protect roots or tooth surfaces that are exposed to the oral cavity, ROD abutment teeth can either be covered by direct filling materials (e.g. resin composite) [11] or by cast copings, commonly made from precious alloys (root caps) [12]. Usually, root caps consist of a customized coping that is cast to a stock post and fits into the root channel of an endodontically treated abutment tooth. The major advantage of a cast coping is that it provides the possibility to solder a precision attachment on top of the coping, leading to additional retention of the ROD [13]. The male part of the precision attachment (e.g. balls, magnets, cylindrical attachments) is soldered on top of the coping, and the corresponding matrix is incorporated into the denture [13, 14]. Depending on the type of attachment, the retention may be individually adjusted according to the patient’s needs, and the matrices can even be replaced when worn out. Some systems offer a selection of various matrix types, each of which provides a distinct retention force. Additionally, there are various designs for the RODs themselves. The two most common ROD designs are “closed design” dentures, which completely cover the remaining abutment teeth and resemble complete dentures from the outside, and “open design” dentures, which have their finish line at the margin of the remaining teeth (Fig. 1) [15]. The open design is said to simplify denture cleaning and provide better saliva circulation [16], which consequently reduces the risk of recurrent caries at the abutment teeth [2]. The closed design is recommended in situations with three or fewer available abutment teeth, as it provides an easy transformation of the ROD to a removable complete denture, in case the extraction of abutment teeth is required [17, 18].

It has been shown that RODs require extensive maintenance to prevent them from technical (e.g. chipping fractures or loss of retention) [19, 20] and/or biological (e.g. recurrent caries or periodontal disease) complications [21, 22]. The factors influencing the number and frequency of complications are not yet conclusively clarified. Therefore, the aim of the present retrospective study was to evaluate patients who had been clinically treated with RODs and to quantify the frequency of biological and technical complications. Furthermore, factors influencing the complication rate were analysed.

## Material and methods

The current clinical retrospective study was approved by the ethics committee of Bern, Switzerland (KEK-BE 268/15). All participants provided their written informed consent.

### Screening

The electronic accounting database of the Department of Reconstructive Dentistry and Gerodontology, School of Dental Medicine, University of Bern, was searched to identify subjects receiving an ROD with cast copings wearing precision attachments between 2002 and 2016 in either the pre- or post-graduate clinics. All identified subjects were contacted by a letter explaining the purpose of the study and were invited for a cost-free clinical examination. After three further attempts by telephone, non-responding subjects were marked unavailable and excluded from the study.

### Clinical examination

Clinical examinations were performed between October 2016 and August 2017 by two post-graduate clinicians who had not been involved in the treatment of any of the included patients. To calibrate inter-examiner reliability, the first five clinical examinations were executed together.

First, study participants completed a general and oral dental history, as well as a dental hygiene questionnaire. Their dental status, including the Decayed, Missing, Filled Teeth index (DMFT), stomatological status, O’Leary plaque index and prosthetic condition, was recorded on standardized forms. Furthermore, the dentures were examined in terms of the ROD design (open vs. closed design) and the type of precision attachment. Additionally, the participants were asked about and the patient records were screened for any biological or technical complications that had occurred since ROD delivery.

### Outcome parameters

The primary outcome parameter was to analyse the frequency of any biological or technical complications since ROD delivery. Biological complications included abutment tooth loss, the presence of denture stomatitis, recurrent caries and abutment tooth fractures. Denture stomatitis was diagnosed by visual inspection and comprised all three types according to the Newton classification: multiple pin-point hyperaemic lesions (type 1), diffuse erythema confined to the mucosa contacting the denture (type 2) and inflammatory papillary hyperplasia (type 3) [23]. Caries lesions were identified visually, using dental loupes with a 3.5-fold magnification and an explorer probe. Only cavitated caries, but not initial demineralization, were recorded.

Technical complications included decementation of the gold coping, coping or post-fractures, loss of retention, loss of the denture matrix and denture base or denture teeth fractures.

Furthermore, the influence of smoking habits (yes vs. no), dental/prosthetic status in the opposing jaw, recall frequency (< 6 months vs. 1–2 years vs. never), the adjuvant use of chlorhexidine (CHX) containing mouthwash or gel (yes vs. occasionally vs. never), the attachment type (ball vs. cylindrical attachment), the number of abutment teeth (≤ 3 vs. > 3) at the day of ROD delivery, the ROD design (open vs. closed), denture cleaning and tooth brushing habits (< 2 vs. ≥ 2/day), denture wearing habits (day only vs. day and night), the denture fabrication setting (pre- vs. post-graduate clinic), the plaque index (≤ 40% vs. > 40%) and the denture location (maxilla vs. mandible), on the frequency of complications was analysed.

### Statistical analysis

For descriptive data analysis, the relative amounts of biological and technical complications, using the total number of RODs as the reference, were calculated. Fisher’s exact test was used to test for independence of complications and categorical factors. Furthermore, odds ratios (OR) and 95% confidence intervals (CI) were calculated, comparing the complication risk in respective groups (e.g. smokers vs. non-smokers). The *p* values and odds ratios were adjusted for the number of abutment teeth per ROD with a logistic regression analysis. Furthermore, ORs, the Youden index and Fisher’s exact test were applied to analyse the influence of ROD age on the complication frequency. Regarding the influence of the type of attachment (ball vs. cylindrical attachment), an additional analysis (Fisher test) with the total number of abutment teeth as a reference was applied. The Mann-Whitney *U* test was used to compare the distribution of the metric variables (plaque index and initial number of abutment teeth) in RODs with and without complications. Finally, a multivariate analysis with a logistic regression was applied, identifying factors that increase the overall complication risk (combined biological and technical), as well as the risk for developing denture stomatitis. All statistical tests were two-sided with a significance level of 5%. The analyses were done with the software Stata/IC 14.2 for Windows.

## Results

### Study sample

A total of 511 former patients treated with RODs could initially be identified from the clinic files. The mean age of those 511 patients was 76.1 ± 11.4 years, wearing RODs with a mean number of 2.5 ± 1.2 abutments. Of those, 246 subjects could not be contacted, and 150 declined the invitation, resulting in a total of 115 subjects with 130 RODs willing to attend a cost-free clinical evaluation. One patient with two RODs was excluded from the study because the original number of abutment teeth could not be identified from the patient file or the ROD itself, resulting in a final study sample of 114 participants with a mean age of 70 years (min-max: 42.9–88.4 years) wearing 128 RODs originally retained by 280 abutment teeth. The mean number of abutments per denture was 2.2 ± 0.9. The mean exposure time of the RODs was 7.9 years (min-max: 3.0–14.9 years). A detailed overview of the patient and OD characteristics is given in Tables 1 and 2.

**Table 1:** Characteristics of included participants

Patient characteristics		*n*	%
Gender	Female	48	42.1
	Male	66	57.9
Age	Min.	42.9	-
	Max.	88.4	-
Smoking habits	Yes	27	23.7
	No	87	76.3
RODs	Total	130	100
	Included	128	98.5
	Excluded	2	1.5
Frequency of tooth brushing	2×/day	82	76.3
	1×/day	44	20.2
	Occasionally	3	2.6
	Never	1	0.9
Frequency of denture cleaning	2×/day	82	64.1
	1×/day	44	34.4
	Occasionally	2	1.6
Use of chlorhexidine	Daily	24	21.1
	Occasionally	18	15.8
	Never	72	63.2
Xerostomia	Yes	38	33.3
	No	76	66.7
Recall frequency	≤ 6 months	56	49.2
	1–2 years	36	31.6
	Never	21	18.4
	Not ascertained	1	0.9
Denture wearing habits	Day	39	30.5
	Day and night	89	69.5

**Table 2:** Characteristics of root cap–retained overdentures (RODs)

Overdenture characteristics		*n*	%
RODs	Maxilla	73	57
	Mandible	55	43
Abutment tooth with post and core	Day of delivery	280	100
	Day of examination	253	90.4
Type of attachment	Cylindrical	20	7.9
	Ball	229	91.5
	Other	4	1.6
ROD design	Open	29	22.7
	Closed	99	77.3
Root caps in situ (years)	Min.	3.0	-
	Max.	14.9	-

### Biological complications

Of the RODs, 46.1% were free of biological complications. At least one biological complication occurred in 53.9% of the RODs. Five types of biological complications could be identified. The most frequent complication per denture was the presence of denture stomatitis (38.3%), followed by the loss of abutment teeth (14.8%), abutment tooth caries (8.6%), fracture of abutment teeth (7.8%) and apical lesions on abutment teeth (3.9%). Table 3 depicts the absolute and relative amounts of biological complications, separated by mandibular and maxillary RODs. In total, 27 of 280 abutment teeth were lost. Factors influencing abutment tooth loss were analysed more comprehensively in another publication. A statistical tendency towards the influence of ROD age on the frequency of biological complications was observed (*p* = 0.051). The mean age of RODs with and without biological complications was 8.5 ± 3.2 years and 7.3 ± 3.5 years, respectively (OR: 1.11). According to the Youden-index, an ROD age of 8 years is the cut-off point: overall biological complications (*p* = 0.008; OR: 2.65) and tooth loss (*p* = 0.004; OR: 4.6) were more frequent in RODs older than 8 years. No influence of the ROD age on other types of biological complications could be demonstrated.

**Table 3:** Biological and technical complications

Per overdenture (*n* = 128)	Man (*n* = 55) *n* (%)	Max (*n* = 73) *n* (%)	*p* value	OR (95% CI) Max vs. Man
Biological complications				
Localized stomatitis	10 (18.2)	20 (27.4)	0.293	1.70 (0.71–4.03) #
Generalized stomatitis	3 (5.5)	9 (12.3)	0.231	2.44 (0.62–9.61) #
Hyperplastic inflammation	2 (3.6)	5 (6.8)	0.698	1.95 (0.36–10.55) #
Loss of abutment teeth	9 (16.4)	10 (13.7)	0.611	0.77 (0.28–2.11) ##
Abutment tooth caries	6 (10.9)	5 (6.8)	0.365	0.56 (0.16–1.97) ##
Fracture of abutment teeth	8 (14.5)	2 (4.1)	*0.016*	0.15 (0.03–0.70 ##
Apical lesions in abutment teeth	2 (3.6)	2 (2.7)	0.979	1.02 (0.16–6.55) ##
Technical complications				
Loss of matrix retention	30 (54.5)	12 (16.4)	0.480	0.77 (0.38–1.56) #
Denture base fracture	8 (14.5)	17 (23.3)	0.264	1.78 (0.70–4.54) #
Root-cap decementation	6 (10.9)	12 (16.4)	0.447	1.61 (0.56–4.62) **##**
Loss of the matrix housing	6 (10.9)	35 (47.9)	0.603	1.45 (0.50–4.22) **##**
Post fracture	4 (7.3)	0 (0)	*0.032*	- **##**

Number (%) of biological and technical complications, comparing mandible (man) and maxilla (max)

^#^Exact Fisher test

##*p* value and OR adjusted for the number of abutment teeth (logistic regression)

The presence of denture stomatitis was significantly higher in the maxilla (*p* = 0.029, OR: 2.32) and in subjects cleaning their RODs less than twice a day (*p* < 0.001, OR: 4.76). The presence of denture stomatitis was also influenced by using CHX-containing products (*p* = 0.036). The lowest frequency of denture stomatitis was found in subjects never using CHX-containing products. Stomatitis tended to occur more frequently in subjects wearing dentures with a closed design; however, this observation was not statistically significant in the participant cohort (*p* = 0.089; OR: 2.32). An overview of the factors influencing the presence of denture stomatitis is given in Table 4. Abutment tooth fractures were more frequent in mandibular RODs (*p* = 0.016) (Table 3).

**Table 4:** Overall stomatitis (localized, generalized and hyperplastic)

Stomatitis	*n* (1)	*n* (2)	*n* (3)	(1)	(2)	(3)	*p* value	OR (95% CI)
Upper (1) vs. lower (2)	73	55	-	34 (46.6.%)	15 (27.3%)	-	*0.029*	0.17 (0.03–0.85)
Smoker (1) vs. non-smoker (2)	97	31	-	36 (371%)	13 (41.9%)	-	0.674	0.82 (0.36–1.87)
Plaque > 40% (1) vs. plaque ≤ 40% (2)	33	94	-	17 (51.5%)	32 (34.0%)	-	0.097	2.06 (0.91–4.67)
Students (1) vs. non-students (2)	83	45	-	33 (39.8%)	16 (35.6%)	-	0.705	1.20 (0.56–2.55)
Denture wearing: day only (1) vs. night and day (2)	39	89	-	11 (28.2%)	38 (42.7%)	-	0.166	1.90 (0.83–4.33)
Cleaning: < 2/day (1) vs. > 2/day (2)	98	30	-	29 (29.6%)	20 (66.7%)	-	*0.000*	4.76 (1.88–12.03)
Design: non-cover (1) vs. cover (2)	29	99	-	7 (24.1%)	42 (42.4%)	-	0.086	2.32 (0.89–6.02)
Abutment-teeth: ≤ 3 (1) vs. > 3 (2)	114	14	-	42 (36.8%)	7 (50.0%)	-	0.389	1.71 (0.56–5.27)
Attachment: ball (1) vs. cylindrical (2)	111	15	-	43 (38.7%)	4 (26.7%)	-	0.411	0.58 (0.17–1.94)
Use of CHX daily (1) vs occasionally (2) vs never (3)	27	22	79	12 (44.4%)	13 (59.1%)	24 (30.4%)	*0.036*	-

Frequency (%) of denture stomatitis, for different subgroups; *p* values (exact Fisher test), odds ratios (OR) and 95% confidence intervals (95% CI)

The Mann-Whitney *U* test confirmed that the plaque index was significantly higher in RODs with abutment tooth caries and denture stomatitis (Table 5). Stomatitis (*p* < 0.001) and abutment tooth caries (*p* = 0.037) were significantly more frequent in denture sites with a plaque index > 40%. No further factors influencing the frequency of biological complications could be identified.

**Table 5:** Plaque index in dentures with and without complications

	Compl	*n*	Mean	SD	*p* value
Biological complications					
Stomatitis	No	78	27.4	19.4	*0.032*
	Yes	49	39.8	29.5	
Localized	No	97	30.7	22.4	0.549
	Yes	30	37.0	30.1	
Generalized	No	115	31.0	24.3	*0.044*
	Yes	12	43.6	24.2	
Hyperpl. Inflammation	No	120	31.4	23.5	0.386
	Yes	7	45.4	37.5	
Loss of abutments tooth	No	109	32.2	23.5	0.737
	Yes	18	32.1	30.1	
Abutment tooth caries	No	116	30.3	23.8	*0.002*
	Yes	11	51.7	23.9	
Apical lesion	No	122	32.7	24.7	0.262
	Yes	5	19.3	13.4	
Fracture of abutment teeth	No	117	32.9	25.0	0.510
	Yes	10	23.8	14.3	
Technical complications					
Root-cap decementation	No	109	32.6	24.9	0.879
	Yes	18	29.4	22.1	
Post-fracture	No	123	32.8	24.5	0.089
	Yes	4	14.3	12.7	
Loss of matrix retention	No	62	27.3	23.8	*0.006*
	Yes	65	36.8	24.3	
Loss of the matrix housing	No	110	31.1	23.1	0.451
	Yes	17	39.2	31.6	
Denture base fracture	No	102	32.3	25.1	0.937
	Yes	25	31.5	21.8	

Plaque index, mean and standard deviations (SD) in dentures with and without complications; *p* values from Mann-Whitney *U* test

### Technical complications

Of all RODs, 31.2% had no technical complications; at least one technical complication occurred in 68.8% of the dentures. Retention loss of the matrices was the most frequently observed complication (50.1%), followed by denture base fractures (19.5%), decementation of the root caps (14.1%), loss of the matrix housing (13.3%) and post-fractures (3.1%). Details on the number of technical complications are given in Table 3. Neither the overall frequency of technical complications (*p* = 0.453) nor of any of the various types of technical complications was significantly influenced by ROD age. The mean age of the RODs with and without technical complications was 8.1 ± 3.3 years and 7.6 ± 3.5 years, respectively (OR: 1.04).

Post-fractures were significantly more frequent in mandibular RODs (*p* = 0.032). Furthermore, post-fractures (*p* = 0.008; Table 6) and decementation of the root caps (*p* = 0.005) were significantly more frequent in RODs worn only during daytime. Post-fractures (*n* = 4) only occurred in RODs with 1 or 2 abutment teeth. However, a higher number of abutment teeth (> 3) correlated significantly with a higher risk of denture base fractures (*p* = 0.031; OR 3.75). The plaque index was significantly higher in dentures that lost matrix retention (*p* = 0.006) (Table 5). No further factors influencing the frequency of technical complications could be identified.

**Table 6:** Post-fractures

Post-fracture	*n* (1)	*n* (2)	(1)	(2)	*p*-value	OR (95% CI)
Upper (1) vs. lower (2)	73	55	0 (0%)	4 (7.3%)	*0.032*	-
Smoker (1) vs. non-smoker (2)	97	31	2 (2.1%)	2 (6.5%)	0.247	0.31 (0.04–2.31)
Plaque > 40% (1) vs. plaque ≤ 40% (2)	33	94	0 (0%)	115	0.572	-
Students (1) vs. non-students (2)	83	45	3 (3.6%)	1 (2.2%)	1.000	1.65 (0.16–16.52)
Denture wearing: day only (1) vs. night and day (2)	39	89	4 (10.3%)	0 (0%)	*0.008*	-
Cleaning: < 2/day (1) vs. > 2/day (2)	98	30	4 (4.1%)	0 (0%)	0.572	-
Design: non-cover (1) vs. cover (2)	29	99	1 (3.4%)	3 (3.0%)	1.000	0.88 (0.09–8.83)
Abutment-teeth: ≤ 3 (1) vs. > 3 (2)	114	14		0 (0%)	1.000	-
Attachment: ball (1) vs. cylindrical (2)	111	15	4 (3.6%)	0 (0%)	1.000	-

Frequency (%) of post-fractures, for different subgroups; *p* values (exact Fisher test), odds ratios (OR), and 95% confidence intervals (95% CI)

### Multivariate analysis

The multivariate logistic regression analysis demonstrated a significantly higher risk for denture stomatitis in subjects brushing their teeth less than twice a day (*p* < 0.001), using CHX-containing products occasionally (*p* = 0.006) or daily (*p* = 0.015) compared with never, and in maxillary RODs (*p* = 0.016). At least one biological or technical complication occurred in 80.5% of all dentures, which means that only 19.5% of the dentures were completely complication-free. Complications were more frequently observed among RODs made and delivered in the pre-graduate clinic (*p* = 0.023). No further factors influencing the overall complication rate could be identified.

## Discussion

This study aimed to analyse the frequency of biological and technical complications in RODs retrospectively in order to identify factors influencing the occurrence of complications. Overall, at least one complication was recorded in 80.5% of the dentures. The most frequently observed biological complication was denture stomatitis. The occurrence of biological complications was significantly higher in subjects who have maxillary dentures, clean their dentures less than twice a day, regularly use CHX-containing products and have dentures with high plaque indices. Additionally, denture stomatitis tended to be more frequent among participants wearing closed-design dentures. The most frequently observed technical complication was the loss of matrix retention. The occurrence of technical complications was significantly higher in mandibular RODs, and when dentures were worn only during the day. While ROD age did not influence the frequency of technical complications, biological complications became more frequent with age: the overall risk of biological complications in RODs older than 8 years was 2.6-fold higher, and the risk of tooth loss was 4.6-fold higher.

The current study is one of only a few studies focusing on complications in RODs. A total of 115 subjects, wearing their denture over a maximum period of 14.9 years (minimum 3.0 years), were clinically evaluated. Furthermore, complications could be retraced from the patient files, as most of the patients were followed up in the Department of Reconstructive Dentistry, University of Bern, after denture delivery. Although RODs are commonly used in Switzerland as an alternative to clasp-retained RPDs, the shortcomings of the extensive tooth preparation and the elective endodontic treatment of the abutment teeth should be considered. Consequently, RODs might better constitute an alternative to implant overdentures.

The overall complication rate of 80.5% (at least one complication per denture) in the present study was high compared with numbers given in similar studies [24]. To correctly interpret this high number of complications, it should be noted that in the current study, loss of retention was regarded as a technical complication, even when the problem could be solved by exchanging a worn matrix. However, when retention loss is not related to the matrix, but to the precision attachment on the root cap, the problem cannot easily be addressed by exchanging the attachment, as the attachment is an inseparable part of the root cap. Usually, these situations require re-fabrication of the ROD, and sometimes even replacement of the natural root by an implant, if the original root cap cannot be separated from the root. Therefore, treating retention loss is one of the main efforts during follow-up. The authors decided to include the loss of retention as a complication, providing a more appropriate estimation of the follow-up procedures in RODs. The number of biological complications was also slightly higher compared with the results of a recently published systematic review [24]. The high complication frequency should be taken into account and discussed with a patient when considering an ROD as a treatment option, not least because of the emerging follow-up costs. However, the complication frequency of RODs is similar to that of other types of RPDs. A study of clasp and conical crown-retained RPDs demonstrated a clinical success rate of 36.6% [25]. It must be noted that those 36.6% still included RPDs with minor repairs; therefore, it can be assumed that the complication-free survival fraction was even lower. In clasp-retained RPDs, the most frequently reported biological complications are the presence of caries (0–32.7%) [26] and inflammation of the soft tissues (35.6%) [27], whereas the most frequent technical complication is the fracture of clasps (16.1%) [26]. The caries frequency in double crown-retained RPDs ranges from 1.8 to 16.4% [26], and the most common technical complication is the partial or complete loss of facings on the secondary crowns (22.2%) [25]. A cut-off point for an increased risk of biological complications, as found in the present study, could not be identified from the current literature on other types of RPDs.

It is very concerning that denture stomatitis was found in approximately one-third of all denture sites. In the scientific literature, even higher frequencies of stomatitis are reported among removable denture wearers [28]. It seems logical that denture stomatitis occurred more frequently in maxillary compared with mandibular RODs, because of the increased size of the soft tissue covering in maxillary dentures, which has previously been demonstrated to be accompanied by a higher risk of denture stomatitis [29]. The potentially higher frequency of denture stomatitis in closed-design dentures, as observed in the current study cohort, supports this theory, although the positive influence of improved salivary access is not yet clear [30, 31]. However, a recently published study has also demonstrated higher failure and biological complication rates, as well as a higher risk of plaque in RODs with a closed relative to an open design [32]. Consequently, an open design seems beneficial for reducing the risk of biological complications in RODs. Interestingly, the beneficial outcomes of an open denture design in terms of plaque levels and inflammatory parameters have also been demonstrated in a study on telescopic crown-retained overdentures [33].

The significantly higher frequency of denture stomatitis in subjects regularly using CHX-containing products is a very interesting finding, especially since most of the current literature encourages the use of CHX due to its anti-candidal effect [34]. A higher risk for stomatitis due to CHX’s side effects has not been reported in the scientific literature. Nevertheless, there are publications demonstrating negative side effects, such as desquamative lesions and soreness while using CHX-containing mouthwashes. The reason for the higher risk of stomatitis could be related to the toxic impact of chlorhexidine on cell membranes, its cytotoxic effect on fibro-, myo-, and osteoblasts, or a shift in the oral microbiome due to the unspecific antiseptic effect of CHX [35–37]. Nevertheless, CHX is reported as a valuable solution for the inhibition of caries [37]. Here, this beneficial effect of CHX-containing products for preventing caries lesions was not observed. However, high plaque indices were correlated with the presence of both recurrent caries and denture stomatitis. Furthermore, denture stomatitis was more frequently found among subjects cleaning their dentures less than twice a day. Summarizing these findings, routine use of CHX-containing products should not be recommended for ROD wearers, whereas adequate denture hygiene seems to be a decisive factor for preventing denture stomatitis and recurrent caries, which are the major biological complications in RODs aside from abutment tooth loss [38].

Abutment tooth and post-fractures were more frequently observed in mandibular compared to maxillary RODs. Additionally, post-fractures only occurred in RODs with fewer than 3 abutment teeth. As fractures are generally regarded to be a direct consequence of overloading, it is not surprising that a low number of abutment teeth results in high forces on these teeth (for example, during chewing) and, consequently, an increased fracture risk [39]. According to the available evidence, another factor for increased fracture risk is a high number of abutments in the opposing jaw [39, 40]. Although the dental status in the opposing jaw was analysed in the current study, no influence on the complication rates was observed. However, increasing the number of abutment teeth appears to come at a cost: denture base fractures were more frequently observed in dentures with > 3 abutment teeth. This might be due to a reduced denture base thickness around abutment teeth, because of space limitations. Since denture base fractures are much easier to treat (for example, by incorporating a denture framework), increasing the number of abutment teeth should still be indicated in order to prevent abutment fractures, especially in mandibular RODs. According to the current results, the minimum number of abutment teeth in an ROD should be three. A recommendation about their distribution based on the present data cannot be provided.

In all cases of root cap decementation, caps could be recemented. In four RODs, a post-fracture of the recemented root cap or other root caps in the same jaw occurred. This may be related to an overloading of the root caps if they were not cemented in the exact same position. Therefore, in cases of root cap decementation, it might be beneficial to remove the matrices and housings from the ROD, and to repolymerize them into the denture intraorally, thereby preventing misfit-induced overloading. Misfit resulting from tooth migration during the night, when RODs are not worn, could also explain the beneficial outcomes in terms of post-fractures and decementation in day-and-night ROD wearers. However, many negative factors, such as increased plaque levels, higher frequencies of gingival inflammation, caries, periodontitis and pneumonia (in elderly subjects), are associated with day-and-night denture wearing [41–43]. Therefore, all included participants were originally instructed to remove their RODs during the night, or to clean the RODs extensively before sleeping, if they wanted to wear them during the night. Considering the positive and negative effects, nocturnal denture wearing should only be recommended in subjects with excellent oral and denture hygiene routines, although the negative effects were not confirmed by the present study. The higher frequency of overall complications in RODs made in the pre-graduate clinic may be caused by several factors, such as the lower level of experience of pre-graduate students, increased time between final impression and ROD delivery allowing for tooth migration, or less stringent follow-up. In terms of the present data, a single factor causing the higher complication rate could not be identified.

The major limitation of the current study is its retrospective design and the high dropout rate. Although patients were asked to provide information on any complication since denture delivery, and all patient records were checked, it cannot be ruled out that the actual number of complications was higher than the reported number. Some patients were followed up in a private practice setting and came back only to participate in the current study, and some patient records could not be located. Furthermore, it would have been interesting to include Kaplan-Meier curves, estimating the likelihood of complications over time. But for many complications, the exact time of occurrence could not be verified, so it was decided not to include such analyses. Additionally, the current study did not analyse periodontal parameters or their influence on the complication frequency.

Nevertheless, the current study provides several key insights on complications in RODs, which have, to the best of the authors’ knowledge, not been described so far.

## Conclusion

Root cap-retained overdentures with precision attachments are a viable treatment option in partially dentate subjects, demonstrating satisfactory clinical outcomes, even over long-term periods. However, frequent technical and biological complications may be expected. The frequency of biological complications is influenced by ROD age, with 8 years being the cut-off point. An open denture design, as well as well-established oral hygiene, can prevent biological complications. Day-and-night denture wearing may reduce the frequency of root cap decementation and post-fractures, but should only be recommended in subjects with excellent oral and denture hygiene routines. A minimum of three abutments seems beneficial for preventing post-fractures. CHX-containing products may foster denture stomatitis if used on a daily basis.
